# Exploring Refinement Strategies for Single Housing of Male C57BL/6JRj Mice: Effect of Cage Divider on Stress-Related Behavior and Hypothalamic-Pituitary-Adrenal-Axis Activity

**DOI:** 10.3389/fnbeh.2021.743959

**Published:** 2021-10-28

**Authors:** An Buckinx, Andries Van Schuerbeek, Jo Bossuyt, Wissal Allaoui, Yana Van Den Herrewegen, Ilse Smolders, Dimitri De Bundel

**Affiliations:** Research Group Experimental Pharmacology, Department of Pharmaceutical Sciences, Center for Neurosciences, Vrije Universiteit Brussel, Brussels, Belgium

**Keywords:** exploratory activity, anxiety, working memory, fear memory, housing conditions

## Abstract

**Introduction:** Single housing of laboratory mice is a common practice to meet experimental needs, or to avoid intermale aggression. However, single housing is considered to negatively affect animal welfare and may compromise the scientific validity of experiments. The aim of this study was to investigate whether the use of a cage with a cage divider, which avoids physical contact between mice while maintaining sensory contact, may be a potential refinement strategy for experiments in which group housing of mice is not possible.

**Methods:** Eight-week-old male C57BL/6JRj mice were single housed, pair housed or pair housed with a cage divider for four (experiment 1) or ten (experiment 2) weeks, after which we performed an open field test, Y-maze spontaneous alternation test, elevated plus maze test, an auditory fear conditioning task, and assessed responsiveness of the hypothalamic-pituitary-adrenal (HPA) axis.

**Results:** Housing conditions did not affect body weight, exploratory activity, anxiety, working memory, fear memory processing or markers for HPA-axis functioning in either experiment 1 or experiment 2. There was an increased distance traveled in mice housed with a cage divider compared to pair housed mice after 4 weeks, and after 10 weeks mice housed with a cage divider made significantly more arm entries in the Y-maze spontaneous alternation test.

**Conclusion:** Taken together, our study did not provide evidence for robust differences in exploratory activity, anxiety, working memory and fear memory processing in male C57BL/6JRj mice that were single housed, pair housed or pair housed with a cage divider.

## Introduction

Rodents comprise the majority of laboratory animals used for scientific purposes, with laboratory mice the most commonly used in biomedical research (European Commission, 2010; Hickman et al., 2017; Carbone, 2021). Animal welfare is a critical factor for animal experimentation, and discomfort that can lead to stress needs to be minimized to ensure the quality and validity of scientific results (Poole, 1997; Baumans, 2005; Kappel et al., 2017). Since mice are social animals (EMA, 2010; Kappel et al., 2017), group housing is the recommended default housing condition for laboratory mice. However, male wild mice live solitary to establish territory, and if successful, in polygamous groups comprising a dominant male, several females and their offspring (Latham and Mason, 2004). This natural social behavior of male mice is challenging to replicate in a laboratory setting. Male mice housed in single sex groups will form despotic hierarchies with dominant-subordinate relationships (Williamson et al., 2016; Kappel et al., 2017). This can consequently lead to inter-individual aggression, and the inability to escape from the aggressor in a confined cage can lead to stress, injuries, pain and even death (Weber et al., 2017; Lidster et al., 2019). Moreover, subordination and social defeat may be associated with detrimental effects on physiology and behavior (Ferrari et al., 1998; Blanchard et al., 2001; Fitchett et al., 2005). These effects seem highly variable and dependent on the social context (Bartolomucci et al., 2001; Kappel et al., 2017; Williamson et al., 2017). Nevertheless, aggression is an important welfare concern and may compromise the quality of scientific results (Van Loo et al., 2003). Therefore, single housing occurs frequently to separate aggressors and the injured mice, although not being the primary procedure to prevent aggression (Kappel et al., 2017; Weber et al., 2017; Lidster et al., 2019; Melotti et al., 2019; Jirkof et al., 2020). Beyond aggression, single housing of laboratory mice could be required for experimental procedures, such as assessment of individual behavioral, metabolic and physiological parameters (Schipper et al., 2018; Manouze et al., 2019), or following specific surgical procedures (Olsson and Westlund, 2007; Manouze et al., 2019). However, the potential benefits of single housing should be weighed against the social needs of mice (Van Loo et al., 2003).

Single housing of mice has not been recommended as a standard procedure (National Research Council, 2011) and is considered to evoke alterations in physiology and behavior (Olsson and Westlund, 2007; Kappel et al., 2017). A higher adrenal gland to body weight ratio and increased stress-induced corticosterone release (Bartolomucci et al., 2003; Berry et al., 2012) have been observed in single housed mice, indicating hypothalamus—pituitary—adrenal (HPA) axis dysregulation (Kolber et al., 2008). However, several studies did not identify significant effects of single housing on fecal or plasma corticosterone levels (Hunt and Hambly, 2006; Arndt et al., 2009; Bailoo et al., 2020; Hohlbaum et al., 2020), and others reported lower basal corticosterone content in urine or plasma of single housed mice (Martin and Brown, 2010; Ieraci et al., 2016; Kamakura et al., 2016). Indeed, in specific contexts individual housing has been reported to be more related to low stress conditions (Brain, 1975). While single housed mice showed less variability in body composition parameters (Nagy et al., 2002), higher visceral adiposity and increased food intake (Sun et al., 2014; Schipper et al., 2018); both lower, higher or equal body weights have been observed compared to group housed mice (Nagy et al., 2002; Võikar et al., 2004; Martin and Brown, 2010; Sun et al., 2014; Kamakura et al., 2016; Pasquarelli et al., 2017; Schipper et al., 2018; Hohlbaum et al., 2020). Single housing has also been shown to increase exploratory activity in novel environments (Palanza et al., 2001; Võikar et al., 2004; Koike et al., 2009; Ieraci et al., 2016; Palanza and Parmigiani, 2017; Pasquarelli et al., 2017), elicit anxiety-like- and depressive-like behavior (Võikar et al., 2004; Koike et al., 2009; Martin and Brown, 2010; Berry et al., 2012; Kalliokoski et al., 2014; Ieraci et al., 2016; Pasquarelli et al., 2017; Liu et al., 2020) and to disrupt cognitive functions (Võikar et al., 2004; Ibi et al., 2008; Liu et al., 2020). However, the effects of single housing may depend on the mouse strain, age, sex, duration of single housing, environmental enrichment, and importantly whether single housed mice were completely isolated from visual, olfactory or auditory contact with other mice or not (Palanza et al., 2001; Võikar et al., 2004; Koike et al., 2009; Martin and Brown, 2010; Kappel et al., 2017; Palanza and Parmigiani, 2017; Bailoo et al., 2020; Hohlbaum et al., 2020).

The 3R principle of refinement in animal experiments (Tannenbaum and Bennett, 2015; Hubrecht and Carter, 2019) aims to ensure that all needs of laboratory animals are met. In this context, it has been proposed that single housing should be limited in time and visual, olfactory and auditory contact with conspecifics should be provided (National Research Council, 2011). For this reason, a cage divider that separates the cage into two compartments while allowing sensory contact between animals may be a viable refinement strategy. Additionally, the reason for using a cage partition is usually employed to maintain a dominant—subordinate polarity in social stress paradigms (D’Amato et al., 2001). Up to now, only a few studies investigated potential benefits of a cage containing a cage divider, separating the cage into two compartments (Rettich et al., 2006; van Loo et al., 2007; Hohlbaum et al., 2020).

Taken together, it remains uncertain whether the application of a cage-divider has a positive impact on the well-being of mice compared to single housing. In this study we specifically aimed to investigate the effects of long-term single housing, pair housing and pair housing with a cage divider on exploratory activity, anxiety, working memory, fear memory processing and HPA-axis activity in C57BL/6JRj mice. Here, single housing was defined as being individually housed in a cage, while visual, auditory and olfactory contact with conspecifics was maintained. We hypothesized that single housing would negatively impact stress-related behaviors and HPA axis activity compared to pair housing and that the negative impact would be reversed by using pair housing with a cage divider.

## Materials and Methods

### Animals

A total of 60 male C57BL/6JRj mice (Janvier laboratories, France) were used in this study. Mice arrived in the animal facility at an age of 7 weeks and were housed under standard temperature (19–25°C) and humidity (30–70% relative humidity) laboratory conditions, receiving regular chow and water *ad libitum*. After arrival, mice were housed in groups of 4–6 mice for 1 week to acclimatize to the animal facility. At the age of 8 weeks, mice were allocated to the experimental groups. During the housing period in the animal facility, mice were kept under a 14/10 h light/dark cycle (lights on at 7:00). One week prior to conducting the first behavioral test, mice were transferred to the laboratory and were maintained in a 12/12 h light/dark cycle (lights on at 7:00) for the remainder of the experiments. Mice were habituated to handling (cupping) by the researchers prior to behavioral testing (for a few minutes/day for 3 days). All procedures were in accordance with local guidelines for animal experiments (Royal Decision 2013-05-29/12, Directive 2010/63/EU), complied the ARRIVE guidelines (Kilkenny et al., 2010) and were approved by the Ethical Committee for Animal Experiments of the Vrije Universiteit Brussel (Ethical approval *n*°: 17-213-4, license date: July 14th 2017).

### Experimental Set-Up

**FIGURE 1:**
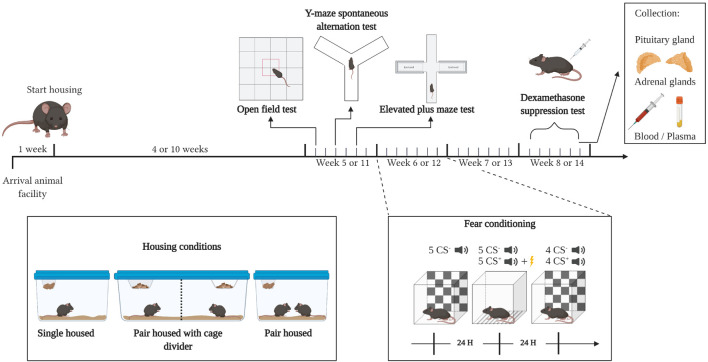
Experimental design. Mice were housed in their respective housing conditions throughout the entire experiment. After four (experiment 1) or ten (experiment 2) weeks of housing, an open field test, Y-maze spontaneous alternation and elevated plus maze test were performed. The next week, mice were subjected to an auditory fear conditioning procedure, and 2 weeks later, mice were subjected to the dexamethasone suppression test, after which they were sacrificed, and plasma, the adrenal glands and pituitary gland were harvested. Starting after the housing period, each area between black (big) indentations represents a week, while each area between gray (small) indentations represents a day. CS^–^,generalization cue; CS^+^, conditioning cue; H, hour. Created with BioRender.com.

Mice were randomly allocated to one of the three experimental groups: single housed (Tecniplast 1264C Eurostandard Type II cages, Italy; 268 × 215 × 141 mm, 370 mm^2^ floor area), pair housed (Tecniplast 1264C Eurostandard Type II cages; 268 × 215 × 141 mm, 370 mm^2^ floor area), or pair housed with a cage divider (Tecniplast 1290D Eurostandard Type III cages with a partition grid and separate wire tops; 425 × 276 × 153 mm, 410 mm^2^ floor area/compartment), dividing the cage into two equal compartments by a transparent Plexiglass partition. The partition was provided with small holes, enabling sensory contact while avoiding physical contact. Both pair housed and mice housed with a cage divider were colony mates and derived from the same group housed condition. Each mouse had access to nesting material (FDA Nestlets, Datesand, United Kingdom), wooden sticks (aspen wood, Tapvei, Estonia), bedding (2HK aspen wood, Tapvei) and a cardboard shelter (Mouse Smart Home, Plexx BV, The Netherlands). Mice were kept in the same housing conditions throughout the experiment and behavioral testing was initiated after 4 weeks in experiment 1 or after 10 weeks in experiment 2. All behavioral experiments were performed during the light phase (Figure 1).

### Behavioral Tests

#### Open Field Test

The open field test (OFT) was performed to assess exploratory activity, locomotor activity and anxiety-like behavior. On the test day (**week 5 or 11**), mice were placed in a corner of the arena, which consists of a square box (60 cm × 60 cm, white opaque floor) surrounded by gray opaque walls (40 cm height) that prevent observations of visual cues from the outside. Mice were left to freely explore the open field for a duration of 10 min. The light intensity in the center of the arena was 80 lux and the arena was swabbed with 70% ethanol between trials. The center of the arena (40 × 40 cm) was defined as the center zone. All trials were recorded with a video tracking system (NCH software, Debut v 2.02^©^, Australia). The total distance traveled and the time spent in the center zone, measurements to evaluate exploratory/locomotor activity and anxiety-like behavior, respectively, were analyzed using a video tracking software (Ethovision 3.0, Noldus, Netherlands).

#### Y-maze Spontaneous Alternation Test

The Y-maze spontaneous alternation test was used to assess spatial working memory. The Y-shaped arena consists of three identical opaque arms (35 cm length × 6 cm width × 20 cm height) that are faced in an angle of 120° from each other. The test is based on the innate tendency of mice to explore novel environments. Depending on their working memory, mice will efficiently alternate between visiting the arms. On the test day (**week 5 or 11**), mice were placed in a random arm (facing the wall) and allowed to freely explore the maze for 8 min. The maze was swabbed with 70% ethanol between trials to eliminate odors and the light intensity was 80 lux. The test was recorded via a video tracking system (NCH software, Debut v 2.02^©^). The spontaneous alternation percentage, a measure for spatial working memory, and the total number of arm entries, a measure for exploratory activity were calculated. An arm entry was defined as having the four paws into that arm. The spontaneous alternation percentage was defined as the total number of alternations (i.e., every time a mouse explored the three arms consecutively) divided by the total number of arm entries minus two, multiplied by 100. Immediate reentries were discounted (Sarnyai et al., 2000; de Bundel et al., 2011; Walrave et al., 2016).

#### Elevated Plus Maze Test

The elevated plus maze (EPM) test was conducted to evaluate anxiety-like behavior. The cross-shaped maze consists of two open arms and two opaque enclosed arms (32.5 cm length × 6 cm width × 16 cm height) with a central open zone (6 cm × 6 cm), elevated to a height of 75 cm from the ground. On the test day (**week 5 or 11**), mice were placed in an enclosed arm facing the wall and were left to freely explore the maze for 5 min. The EPM was swabbed with 70% ethanol between trials and the light intensity in the open center of the maze was 90 lux. The time spent in the open arms and the total distance traveled, measurements to evaluate anxiety-like behavior and exploratory/locomotor activity, respectively, were recorded and analyzed using a video tracking software (Ethovision 3.0, Noldus).

#### Fear Conditioning

The auditory fear conditioning paradigm was used to study fear memory formation as previously described (De Bundel et al., 2016; Van Schuerbeek et al., 2021). The experiments were carried out in a fear conditioning apparatus containing a test box (17 cm width × 17 cm length × 24 cm height) placed within a soundproof chamber (Isolation Cubicle 46000-590, Ugo Basile, Italy). Two different context configurations were used (context A: checkered walls, white rubber ground floor, swabbed with 1–3% hospital antiseptic concentrate, 15 lux light intensity; context B: gray walls, metal grid, swabbed with 1% acetic acid, 125 lux light intensity, plexiglass plate on top). Two different tones were presented during the fear conditioning procedure (2.5 or 7.5 kHz, 80 dB, 30 s), semi-randomly assigned as generalization cue (CS^–^) or conditioning cue (CS^+^). Tone frequency was counterbalanced across the experimental groups.

On day 1 of the procedure (**week 6 or 12**), mice were placed in context A for a habituation session. After 2 min of acclimation to the test box (HAB), mice were exposed to five presentations of CS^–^ (2.5 or 7.5 kHz, 80 dB, 30 s). The interval between CS^–^ presentations was randomized between 20 and 120 s. The next day, discriminative auditory fear conditioning was performed in context B. After 2 min of acclimation to the test box (HAB), mice were exposed to five pairings of CS^+^ (2.5 or 7.5 kHz, 80 dB, 30 s) with an unconditioned stimulus (US, 0.6 mA electric foot shock, 2 s, coinciding with the last 2 s of CS^+^ presentation). The CS^–^ cue was presented intermittently, preceding each CS^+^—US pairing, but never coinciding with the US. The interval between CS^–^ and CS^+^ presentations was randomized between 20 and 120 s. On day 3, fear memory was assessed in a fear retrieval test in context A. Following 2 min of acclimation to the test box (HAB), CS^–^ and CS^+^ were presented in subsequent blocks of four tone presentations, with a 20–120 s inter-tone interval.

Freezing behavior during HAB, CS^–^ and CS^+^ presentation was analyzed using an automated video monitoring system (Ethovision XT software, RRID:SCR_000441, Noldus). Freezing was defined as the difference of pixels (max. 0.3%) between two consecutive frames during 1 s or more. Additionally, the integrated data was re-analyzed by a blinded observer and corrected for false positives. Time frames that were considered by the software erroneously as freezing, were subtracted manually from the total freezing time. The average time spent freezing during the acclimation period prior to tone presentation (HAB) was used as a measure for contextual fear.

### Dexamethasone Suppression Test

Mice received an intraperitoneal (i.p.) injection of dexamethasone (0.05 mg/kg; body volume 10 mL/kg; Bio-Techne, Minneapolis, MN, United States) dissolved in 1% dimethylsulfoxide (DMSO; Honeywell Fluka) or 1% DMSO in saline (0.9% w/v of NaCl, Baxter, Belgium) between 10:00 and 11:00 a.m. (**week 8 or 14**). Six hours later, mice were sacrificed by administration of an overdose of sodium pentobarbital (Dolethal^®^, Vetoquinol, Belgium) diluted in 0.9% saline. Upon loss of reflexes, cardial blood was collected and placed in heparinized tubes. The total procedure from opening the cage to blood collection was performed in less than 3 min, in order to avoid stress- generated alterations in endogenous plasma corticosterone levels. Blood samples were centrifuged (4°C, 2,500 *g*, 15 min) and plasma was stored at –20°C upon further analysis.

### Organ Weights

After blood collection, the pituitary gland and adrenal glands were dissected and immediately weighed on an analytical scale. The weights of the pituitary and adrenal glands were used as indicators of chronic stress (Everds et al., 2013) and normalized to body weight of mice.

### Enzyme-Linked Immunosorbent Assay

Plasma corticosterone concentrations were assessed using an enzyme-linked immunosorbent assay (ELISA; Abcam, #108821, RRID:AB_2889904, United Kingdom). The protocol was performed according to the manufacturer’s instructions, and plasma samples were diluted 1/50 in the recommended buffer.

### Statistical Analyses

Statistical analysis was performed using Graphpad Prism software 6.0. Values are expressed as mean ± SEM and alpha was set at 0.05.

Ordinary One-Way ANOVA and Repeated Measures (RM) Two-Way ANOVA were performed for statistical analysis. Tukey’s multiple comparison test or Sidak’s multiple comparison test (for *a priori* specified comparisons between dexamethasone and vehicle treated mice) were used for *post hoc* analysis. Experimenters were blinded for housing conditions or dexamethasone treatment at the time of data analysis. Mean values from the performed tests are summarized in Supplementary Table 1.

## Results

### Body Weight

**FIGURE 2:**
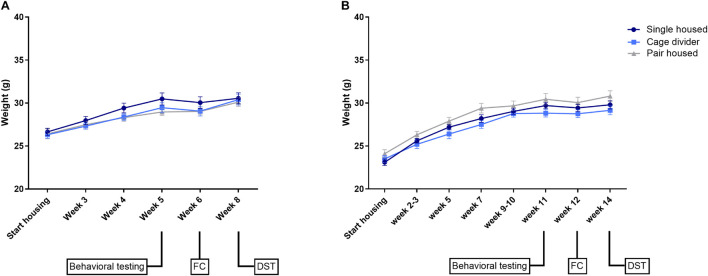
Absolute body weights of mice that are single housed, pair housed and pair housed with a cage divider. **(A)** Absolute body weights in experiment 1. **(B)** Absolute body weights in experiment 2. Data are presented as mean ± SEM. *n* = 10 mice/housing condition. Statistical analysis: Repeated measures Two-way ANOVA. FC, fear conditioning; DST, dexamethasone suppression test.

In both experiments, absolute body weights were not significantly different between housing conditions but increased over time [Exp. 1: Interaction: *F*_(10_, _135)_ = 1.376, *P* = 0.1977; Time effect: *F*_(5_, _135)_ = 111.6, *P* < 0.0001; Housing effect: *F*_(2_, _27)_ = 0.8568, *P* = 0.4357; Figure 2A—Exp. 2: Interaction: *F*_(14_, _189)_ = 1.659, *P* = 0.0674; Time effect: *F*_(7_, _189)_ = 371.1, *P* < 0.0001; Housing effect: *F*_(2_, _27)_ = 2.377, *P* = 0.1120; Figure 2B]. Similarly, normalized body weights were not significantly different between housing conditions in either experiment (Supplementary Figure 1).

### Open Field Test

**FIGURE 3:**
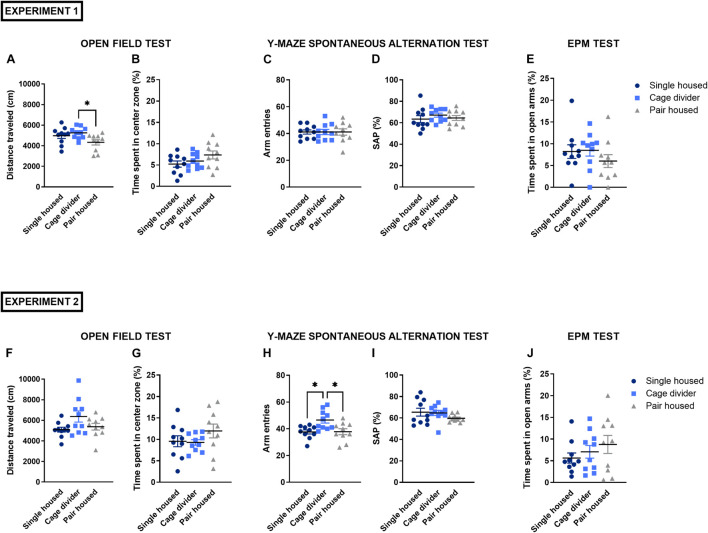
Behavioral testing in mice that are single housed, pair housed and pair housed with a cage divider. **(A)** Total distance traveled in the OFT (10 min) in experiment 1. **(B)** Time spent in the center zone of the OFT in experiment 1. **(C)** Number of arm entries in the Y-maze spontaneous alternation test (8 min) in experiment 1. **(D)** Spontaneous alternation percentage in the Y-maze test in experiment 1. **(E)** Time spent in the open arms of the EPM (5 min) in experiment 1. **(F)** Total distance traveled in the OFT in experiment 2. **(G)** Time spent in the center zone of the OFT in experiment 2. **(H)** Number of arm entries in the Y-maze spontaneous alternation test in experiment 2. **(I)** Spontaneous alternation percentage in the Y-maze test in experiment 2. **(J)** Time spent in the open arms of the EPM in experiment 2. Data are presented as mean ± SEM. *n* = 10 mice/housing condition. Statistical analysis: Ordinary One-way ANOVA with Tukey’s multiple comparisons test for comparisons between single housed, pair housed and mice housed with a cage divider. ^∗^*P* < 0.05. OFT, open field test; EPM, elevated plus maze test; SAP, spontaneous alternation percentage.

The total distance traveled, an indication for exploratory activity, was statistically different between housing conditions in experiment 1 [*F*_(2_, _27)_ = 3.892, *P* = 0.0327; Figure 3A], while in experiment 2 only a trend was observed [*F*_(2_, _27)_ = 3.001, *P* = 0.0666; Figure 3F]. In experiment 1, mice housed with a cage divider had an increased total distance traveled compared to pair housed mice (*P* = 0.0295). The time spent in the center zone, a measure to evaluate anxiety-like behavior, was not significantly different between housing conditions in experiment 1 [*F*_(2_, _27)_ = 2.064, *P* = 0.1465; Figure 3B] and experiment 2 [*F*_(2_, _27)_ = 1.403, *P* = 0.2633; Figure 3G]. These latter parameters were also assessed in the time intervals of 0–5 and 5–10 min (Supplementary Figures 2, 3).

### Y-maze Spontaneous Alternation Test

The spontaneous alternation percentage was not significantly affected by housing conditions in experiment 1 [*F*_(2_, _27)_ = 0.5695, *P* = 0.5725; Figure 3D] and experiment 2 [*F*_(2_, _27)_ = 1.396, *P* = 0.2649; Figure 3I]. Similarly, housing conditions did not affect the number of arm entries in experiment 1 [*F*_(2_, _27)_ = 1.000, *P* = 0.3811; Figure 3C], while in experiment 2 mice housed with a cage divider had a significant increase in the number of arm entries [*F*_(2_, _27)_ = 6.256, *P* = 0.0059; Figure 3H] compared to single- (*P* = 0.0122) and pair housed mice (*P* = 0.0144).

### Elevated Plus Maze Test

The time spent in the open arms of the EPM did not significantly differ between housing conditions in both experiment 1 [*F*_(2_, _27)_ = 0.8537, *P* = 0.4370; Figure 3E] and experiment 2 [*F*_(2_, _27)_ = 0.9514, *P* = 0.3988; Figure 3J]. Similarly, the total distance traveled was unaffected by housing conditions in either experiment 1 [*F*_(2_, _27)_ = 0.0283, *P* = 0.9721; Supplementary Figure 4A] and experiment 2 [*F*_(2_, _27)_ = 0.9921, *P* = 0.3839; Supplementary Figure 4B].

### Fear Conditioning

**FIGURE 4:**
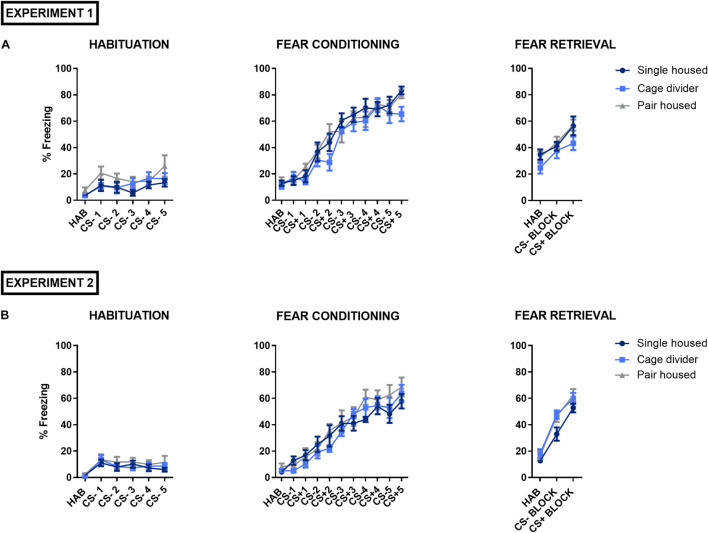
Auditory fear conditioning in mice that are single housed, pair housed and pair housed with a cage divider. The graphs illustrate the freezing responses of mice during the acclimation period (HAB) or evoked by cue presentation (CS^–^, CS^+^) during the habituation session to CS^–^, auditory fear conditioning and fear retrieval test. **(A)** Overview of the fear conditioning procedure in experiment 1. **(B)** Overview of the fear conditioning procedure in experiment 2. Data are presented as mean ± SEM. *n* = 10 mice/housing condition. Freezing scores during CS^–^ and CS^+^ presentation are presented as the average of four tone presentations during the fear retrieval test. Statistical analysis: Repeated measures Two-Way ANOVA.

We studied the effects of housing conditions on fear memory processing in mice using a discriminative auditory fear conditioning procedure. Mice learned to distinguish between two auditory cues, associated (CS^+^) or not (CS^–^) with an electric shock (US). During the habituation session to CS^–^ on day 1, we did not observe a significant housing effect or interaction but a significant cue effect, showing that freezing increased slightly upon repeated cue presentations, in both experiment 1 [Interaction: *F*_(10_, _135)_ = 0.6577, *P* = 0.7617; Cue effect: *F*_(5_, _135)_ = 5.203, *P* = 0.0002; Housing effect: *F*_(2_, _27)_ = 2.895, *P* = 0.0726; Figure 4A] and experiment 2 [Interaction: *F*_(10_, _135)_ = 0.3455, *P* = 0.9667; Cue effect: *F*_(5_, _135)_ = 7.634, *P* < 0.0001; Housing effect: *F*_(2_, _27)_ = 0.5704, *P* = 0.5720; Figure 4B]. During fear conditioning on day 2, no significant housing effect or interaction but a significant cue effect, with increased freezing upon repeated cue presentations, was observed in experiment 1 [Interaction: *F*_(20_, _270)_ = 0.9189, *P* = 0.5634; Cue effect: *F*_(10_, _270)_ = 74.09, *P* < 0.0001; Housing effect: *F*_(2_, _27)_ = 1.972, *P* = 0.1587; Figure 4A] and experiment 2 [Interaction: *F*_(20_, _270)_ = 0.7927, *P* = 0.7220; Cue effect: *F*_(10_, _270)_ = 55.05, *P* < 0.0001; Housing effect: *F*_(2_, _27)_ = 0.8800, *P* = 0.4264; Figure 4B]. During fear retrieval on day 3, no significant housing effect or interaction but a significant cue effect was observed in experiment 1, indicating that mice discriminated between auditory cues [Interaction: *F*_(4_, _54)_ = 0.4281, *P* = 0.7877; Cue effect: *F*_(2_, _54)_ = 18.37, *P* < 0.0001; Housing effect: *F*_(2_, _27)_ = 2.463, *P* = 0.1041; Figure 4A]. Indeed, mice displayed high freezing levels during CS^+^ presentation compared to CS^–^ (*P* = 0.0063) and significantly higher freezing was also observed during CS^–^ presentation compared to HAB (*P* = 0.0166), suggesting a certain degree of fear generalization. In experiment 2, a significant housing effect but no significant interaction was observed, while the significant cue effect similarly indicated that all mice discriminated between auditory cues [Interaction: *F*_(4_, _54)_ = 0.7824, *P* = 0.5416; Cue effect: *F*_(2_, _54)_ = 126.3, *P* < 0.0001; Housing effect: *F*_(2_, _27)_ = 3.956, *P* = 0.0311; Figure 4B]. *Post hoc* analysis, comparing the housing conditions, showed a trend but no significant difference in freezing between single housed and pair housed mice with a cage divider (*P* = 0.0538) or between single housed- and pair housed mice (*P* = 0.0560) during the fear retrieval test.

### Dexamethasone Suppression Test

The dexamethasone suppression test was performed at the end of the experiment to investigate responsiveness of the HPA axis (Butler et al., 2014). Mice without functional impairments of the HPA-axis will show a decrease in plasma corticosterone following injection of the corticosteroid receptor agonist dexamethasone (Kolber et al., 2008; Butler et al., 2014). Reduced dexamethasone suppression therefore indicates an impaired negative feedback loop (Farooq et al., 2018).

**FIGURE 5:**
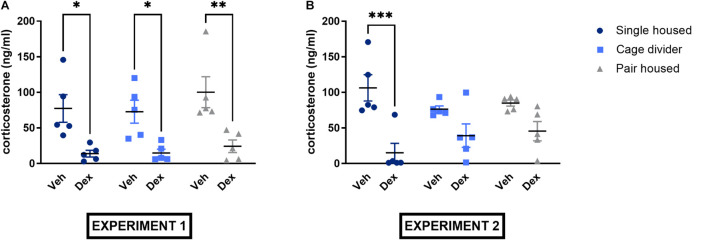
Plasma corticosterone concentrations of single housed, pair housed and pair housed mice with a cage divider subjected to the dexamethasone suppression test. **(A)** Plasma corticosterone levels from the dexamethasone suppression test of experiment 1. **(B)** Plasma corticosterone levels from the dexamethasone suppression test of experiment 2. Data are presented as mean ± SEM. *n* = 5 mice/treatment group. Statistical analysis: Ordinary Two-Way ANOVA with Sidak’s multiple comparisons test for comparisons between mice receiving dexamethasone and vehicle. ^∗^*P* < 0.05, ^∗∗^*P* < 0.01, ^∗∗∗^*P* < 0.001. Dex, dexamethasone; Veh, vehicle.

In experiment 1, no significant housing effect or interaction but a significant treatment effect was observed [Interaction: *F*_(2_, _24)_ = 0.2055, *P* = 0.8157; Treatment effect: *F*_(1_, _24)_ = 31.62, *P* < 0.0001; Housing effect: *F*_(2_, _24)_ = 1.002, *P* = 0.3821; Figure 5A]. Indeed, *post hoc* analysis showed significantly lower plasma corticosterone levels in all mice receiving dexamethasone compared to vehicle (single housed: *P* = 0.0135; pair housed: *P* = 0.0030; cage divider: *P* = 0.0256). Similarly, no significant housing effect or interaction but a significant treatment effect was observed in experiment 2 [Interaction: *F*_(2_, _24)_ = 2.769, *P* = 0.0828; Treatment effect: *F*_(1_, _24)_ = 27.92, *P* < 0.0001; Housing effect: *F*_(2_, _24)_ = 0.1666, *P* = 0.8475; Figure 5B]. In the *post hoc* analysis, dexamethasone significantly lowered plasma corticosterone only in single housed mice (*p* = 0.0001) but not in pair housed mice (*p* = 0.1209) and mice housed with a cage divider (*p* = 0.1513) (Figure 5B). In addition, basal plasma corticosterone levels in vehicle-treated mice did not differ significantly between housing conditions in either experiment [Interaction: *F*_(2_, _24)_ = 0.9980, *P* = 0.3834; Duration of housing: *F*_(1_, _24)_ = 0.2071, *P* = 0.6532; Housing effect: *F*_(2_, _24)_ = 0.8340, *P* = 0.4465].

### Organ Weights

**FIGURE 6:**
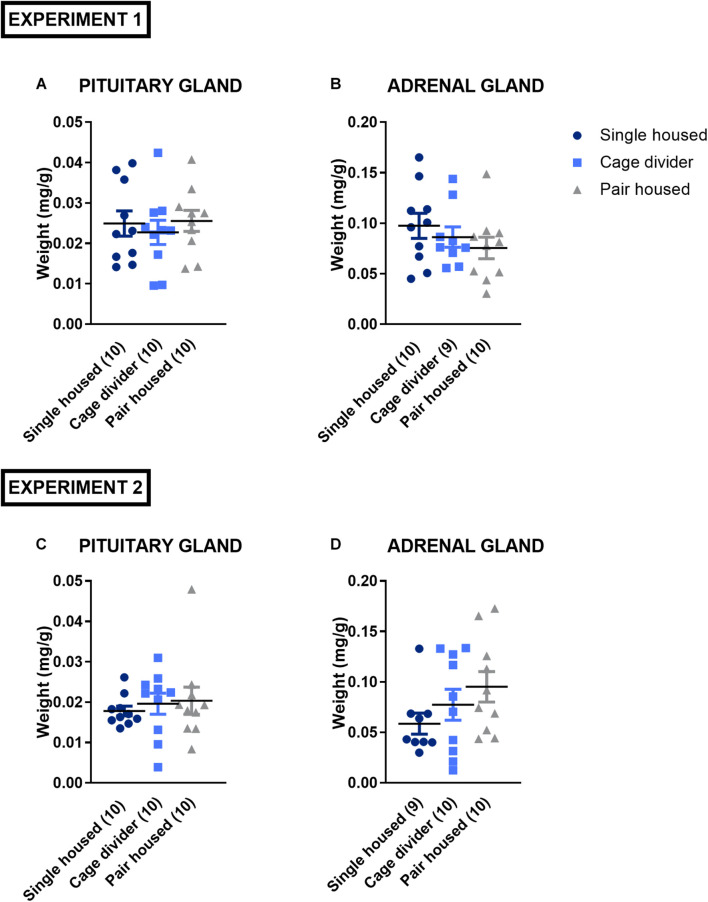
Weights of pituitary and adrenal glands normalized to body weight from mice that were single housed, pair housed and pair housed with a cage divider. **(A)** Normalized pituitary gland weight of experiment 1. **(B)** Normalized adrenal gland weight of experiment 1. **(C)** Normalized pituitary gland weight of experiment 2. **(D)** Normalized adrenal gland weight of experiment 2. Data are presented as mean ± SEM. *n* = 9–10 mice/housing condition. Statistical analysis: Ordinary One-way ANOVA.

Chronic stress might evoke alterations in adrenal glands- and pituitary gland weight (Ulrich-Lai et al., 2006). No significant effect of housing was observed on normalized pituitary gland weight in experiment 1 [*F*_(2_, _27)_ = 0.2602, *P* = 0.7728; Figure 6A] and experiment 2 [*F*_(2_, _27)_ = 0.2594, *P* = 0.7734; Figure 6C]. Similarly, normalized bi-adrenal gland weights were unaffected by housing conditions in both experiment 1 [*F*_(2_, _26)_ = 0.9993, *P* = 0.3818; Figure 6B] and experiment 2 [*F*_(2_, _26)_ = 1.662, *P* = 0.2092; Figure 6D].

## Discussion

Understanding the impact of housing conditions on the well-being of laboratory mice is pivotal for the quality and reproducibility of scientific results (Poole, 1997; Baumans, 2005; Kappel et al., 2017). While aggression may occur in group housed male mice, single housing may cause negative effects on physiology and behavior (Olsson and Westlund, 2007; Kappel et al., 2017). Moreover, single housing is often required for experimental reasons (Jirkof et al., 2012; Kappel et al., 2017; Schipper et al., 2018; Manouze et al., 2019), although there are more appropriate solutions to reduce aggression. Indeed, smart cage design and providing enrichment to increase environmental complexity are considered more appropriate husbandry practices (Kappel et al., 2017; Lidster et al., 2019). In the present study, we explored the use of a cage divider, allowing sensory contact but avoiding physical contact, as potential refinement strategy to overcome the putative negative effects associated with single housing or group housing of male C57BL6/JRj mice. Indeed, single housing is not always associated with negative effects (Brain, 1975) and is currently a hotly debated topic. However, in our experiment, individual housing of male C57BL6/JRj mice did not negatively affect the studied parameters compared to pair housing. We compared the effects on exploratory activity, anxiety, working memory, fear memory processing and HPA-axis responsiveness after four (experiment 1) or ten (experiment 2) weeks of pair housing with a cage divider with single housing and pair housing.

We did not observe a significant effect of housing conditions on anxiety-like behavior, evaluated by time spent in the center zone of the OFT and time spent in the open arms of the EPM, in both experiment 1 and 2. These data are in line with other studies, reporting no differences or even reduced anxiety in single housed C57BL/6J mice compared to group housed mice (Brain, 1975; Võikar et al., 2004; Lopez and Laber, 2015) or pair housed mice (Lad et al., 2010; Pan-Vazquez et al., 2015). Similarly, a recent study showed no significant effect of housing mice with a cage divider on anxiety-related behavior (Hohlbaum et al., 2020). Nevertheless, other studies have suggested that single housed mice are more anxious than group housed mice (Chourbaji et al., 2005; Kwak et al., 2009; Berry et al., 2012; Demuyser et al., 2016; Ieraci et al., 2016; Hebda-Bauer et al., 2019). These conflicting results may be dependent on a variety of factors. Indeed, the strain of mice plays an important role in sensitivities to specific experimental paradigms (van Gaalen and Steckler, 2000; Lad et al., 2010). Additionally, the duration of housing appears to influence anxiety, with shorter housing duration showing increased anxiety-like behavior in single housed mice compared to group housed mice (Kwak et al., 2009; Berry et al., 2012; Demuyser et al., 2016; Ieraci et al., 2016; Hebda-Bauer et al., 2019). Also cage enrichment appears to be a critical factor in stress levels of mice (Chourbaji et al., 2005; Lopez and Laber, 2015). Order effects of testing have been shown to affect the stress response in female mice that were socially housed (Arndt et al., 2009). Although this effect was not apparent in males (Arndt et al., 2009), we alternated the within-cage testing order of mice to avoid possible effects on endocrinological parameters (Theil et al., 2020).

We found an increased distance in the OFT after housing with cage divider for 4 weeks compared to pair housing without cage divider, indicating increased exploratory activity (Crusio, 2001). Additionally, after 10 weeks of housing mice with a cage divider, we observed a higher amount of arm entries in the Y-maze task compared to single housing or pair housing. Observed transient differences may be related to differences in cage area per mouse between the different experimental groups, although a previous study showed that cage size or a reduction in cage size did not affect locomotor activity in the OFT after 4 weeks of housing (Kwak et al., 2009). However, this finding was not present in other novel environments such as the EPM (Supplementary Figure 4), or the fear conditioning chamber (Supplementary Figure 5). Therefore, the results should be interpreted with caution and do not indicate a robust effect of the investigated housing condition on exploratory activity. The available literature similarly does not demonstrate a consistent effect. While a higher exploratory activity was previously observed in single housed C57BL/6J mice compared to group housed mice (Lad et al., 2010; Ieraci et al., 2016; Pasquarelli et al., 2017), others studies reported no differences in exploratory activity (Võikar et al., 2004; Berry et al., 2012; Demuyser et al., 2016). One previous study showed decreased exploratory behavior of pair housed mice with a divider in the social interaction test (Hohlbaum et al., 2020).

We did not observe an effect of housing conditions on the spontaneous alternation percentage in the Y-maze task, a test typically used to assess spatial working memory (Mugwagwa et al., 2015; Walrave et al., 2016; Bak et al., 2017; Kraeuter et al., 2019). This is in line with a previous study showing no deficits in spatial working memory between single- and group housed male C57BL/6J mice (Võikar et al., 2004). The Y-maze spontaneous alternations task gives limited insight in spatial working memory (Hughes, 2004), and therefore the effects of housing conditions on spatial memory should be further scrutinized in other behavioral tasks. Indeed, spatial learning and memory deficits were reported in single housed mice compared to group housed mice in more complex task such as the Morris water (Cao et al., 2017; Liu et al., 2020). Additionally, we did not observe significant effects of housing conditions on fear processing in a discriminative auditory fear conditioning task. Previous findings showed reduced freezing in C57BL/6JOlaHsd mice and DBA mice after 7 weeks of single housing compared to group housing (Holmes et al., 2002; Võikar et al., 2004). While it has been described previously that social interaction between cage mates may facilitate fear memory consolidation, through aversive fear-related odorants or pheromones, we did not observe such effect (Arakawa, 2018). Nevertheless, it can be further explored whether housing conditions could affect contextual fear conditioning and fear extinction in mice. Indeed, deficits in extinction were observed in single housed rats (Skelly et al., 2015) and mice (Pibiri et al., 2008).

Overall, we did not observe major effects of housing conditions on the behavioral parameters assessed in this study. Similarly, we found no significant differences in body weight, corticosterone levels, and adrenal gland- and pituitary gland weight of the mice. Body weight increased significantly over time irrespective of housing conditions in experiments 1 and 2. A previous study showed alterations in body composition but no differences in body weight between single- and group housed male C57BL/6 mice (Sun et al., 2014). Conversely, lower absolute body weight was previously described in single housed male C57BL/6J mice compared to group housed mice (Nagy et al., 2002; Võikar et al., 2004; Pasquarelli et al., 2017) or pair housed siblings (Lad et al., 2010). Furthermore, a previous study showed lower normalized body weight in both pair housed C57BL/6JRJ mice with a cage divider and single housed mice compared to group housing (Hohlbaum et al., 2020). These inconsistent findings may be explained by differences in study design, using group housed mice with 3–5 mice per cage as controls, the use of distinct C57BL/6J sub strains or that mice were housed at a different age and/or for a different duration in these studies (Nagy et al., 2002; Võikar et al., 2004; Lad et al., 2010; Pasquarelli et al., 2017; Hohlbaum et al., 2020). Moreover, one study also reported the decrease in body weight in single housed mice to be transient (Pasquarelli et al., 2017).

At the end of both experiments, we tested whether the housing conditions induced alterations in plasma corticosterone concentrations, the main glucocorticoid produced in mice and a biomarker of stress (Hunt and Hambly, 2006; Arndt et al., 2009; Gong et al., 2015). We could not detect significant effects of housing conditions on basal plasma corticosterone concentrations. This is in line with previous observations, showing no increase in corticosterone levels in single housed C57BL/6J or MF1 mice compared to group housed mice (Hunt and Hambly, 2006; Arndt et al., 2009; Kamakura et al., 2016; Hohlbaum et al., 2020). Plasma corticosterone levels were assessed 6 h after receiving an intraperitoneal injection of either dexamethasone or saline, in order to determine whether the negative feedback loop of the HPA-axis was impaired (Ridder et al., 2005; Kolber et al., 2008). In experiment 1, dexamethasone effectively suppressed plasma corticosterone levels in all housing conditions. In experiment 2, the suppression of plasma corticosterone following dexamethasone administration did not reach statistical significance in pair housed mice with or without cage divider but a clear trend was observed. Additionally, no significant differences were observed in bi-adrenal- and pituitary gland weight in both experiments, further indicating a lack of HPA-axis dysregulation (Ulrich-Lai et al., 2006; Everds et al., 2013).

A handful of studies have investigated effects of a cage divider on animal welfare so far. One study using a partial cage divider was shown to mitigate aggression in group housed Balb/c male mice compared to standard cages (Tallent et al., 2018). However, housing mice with a cage divider after surgery was not more beneficial compared to housing mice individually (van Loo et al., 2007), and even induced distress and impaired well-being of vasectomized Hsd:NMRI males (Rettich et al., 2006). Yet another study showed no differences in burrowing performance, social interaction, anxiety or stress hormone concentrations between adult male C57BL/6JRj mice that were single housed, group housed or housed with a cage divider for 8 weeks (Hohlbaum et al., 2020). Based on previous studies and our study it appears that improving welfare via housing mice with a cage divider is highly context-dependent. Indeed, it mitigates aggression (Tallent et al., 2018), but impairs welfare after surgical procedures (Rettich et al., 2006) and is not beneficial when there is no apparent reason to single house mice (Hohlbaum et al., 2020), as was the case in our study. Additionally, it is important to consider that the C57BL/6J strain shows lower levels of aggression compared to some other strains (Parmigiani et al., 1999; Lidster et al., 2019).

Our study has a few limitations. First of all, we used male mice as the prevalence of aggression in group housed mice, which is often used a reason to single house mice, is almost completely restricted to male mice (Theil et al., 2020). However, the effects of single housing in female mice should also be considered when single housing is required for experimental purposes, given female mice are more socially active than males (Palanza et al., 2001; An et al., 2011; Palanza and Parmigiani, 2017; Arakawa, 2018). Additionally, female mice are increasingly used in biomedical research. Therefore, the effects of single housing in female mice warrants further investigation. Indeed, differences in anxiety and memory were previously observed between single housed and group housed female C57BL/6J mice (Martin and Brown, 2010; Kulesskaya et al., 2011; Liu et al., 2020). As the C57BL/6 mouse is the most widely used mouse strain in biomedical research (Sarsani et al., 2019), we decided to use this strain. However, genetic background of the used mouse strain seems to play a critical role in anxiety and aggression (Parmigiani et al., 1999; Võikar et al., 2004; Lad et al., 2010; Lidster et al., 2019). Actually, the C57BL/6 mouse strain shows low levels of aggression and exhibits moderate anxiety levels under natural conditions (Parmigiani et al., 1999; Sartori et al., 2011; Lidster et al., 2019). This may lead to an underestimation of potential effects on stress and anxiety, account for conflicting results of housing on anxiety and memory between our study and the available literature (Võikar et al., 2004; Lad et al., 2010), and limits extrapolation of the results to other mouse strains. Finally, the C57BL/6J is a mouse strain that seems capable to rapidly adapt to housing conditions (Melotti et al., 2019), which may explain why we could not observe notable differences in our study. Moreover, it should be noted that in our experiments, all mice were provided with minimal enrichment under the form of wooden gnawing sticks, nest material, and a shelter. This may have contributed to the lack of significant differences between pair housed and single housed mice.

Taken together, our study did not provide evidence for robust differences in exploratory activity, anxiety, working memory and fear memory processing in male C57BL/6JRj mice that were single housed, pair housed or pair housed with a cage divider. As such we did not confirm the hypothesis that single housing would negatively impact stress-related behaviors and HPA-axis activity compared to pair housing and that this negative impact would be reversed by using pair housing with a cage divider. Importantly, our conclusion that there was no significant indication of stress following single housing in male C57BL/6JRj mice is not intended to extend more generally to other strains. Moreover, improving welfare via housing mice with a cage divider is highly context-dependent and may thus only be beneficial in specific situations where single-housing has negative effects. Although pair housing with a cage divider may be a solution for single from an ethical point of view, further research is necessary to determine in which contexts this housing system really refines the well-being of laboratory male mice.

## Significance to the Field

Single housing of mice is considered to negatively affect animal welfare and is a major topic in laboratory animal legislation and frameworks. We investigated whether pair housing of mice with a cage divider could offer a refinement for single housing in male C57BL/6JRj mice. We investigated the consequences of long-term exposure to these specific housing conditions for a range of stress-related behaviors and HPA-axis parameters, but we found no evidence for robust differences between the investigated housing conditions in male C57BL/6JRj mice.

## Data Availability Statement

The raw data supporting the conclusions of this article will be made available by the authors, without undue reservation.

## Ethics Statement

The animal study was reviewed and approved by the Ethical Committee for Animal Experiments of the Vrije Universiteit Brussel.

## Author Contributions

AB, AV, YV, IS, and DD: conceptualization and methodology. AB, AV, and JB: validation. AB, AV, and WA: investigation. IS and DD: resources, supervision, and funding acquisition. AB, AV, JB, and DD: writing—original draft preparation. AB, AV, WA, YV, IS, and DD: writing—review and editing. AB and AV: visualization and project administration. All authors contributed to the article and approved the submitted version.

## Conflict of Interest

The authors declare that the research was conducted in the absence of any commercial or financial relationships that could be construed as a potential conflict of interest.

## Publisher’s Note

All claims expressed in this article are solely those of the authors and do not necessarily represent those of their affiliated organizations, or those of the publisher, the editors and the reviewers. Any product that may be evaluated in this article, or claim that may be made by its manufacturer, is not guaranteed or endorsed by the publisher.

## Abbreviations

ANOVA: Analysis of variances
ARRIVE: Animal Research: Reporting of *In Vivo* Experiments
CS^–^: generalization cue
CS^+^: conditioning cue
dB: decibel
DMSO: dimethylsulfoxide
DST: dexamethasone suppression test
ELISA: enzyme-linked immunosorbent assay
EPM: Elevated plus maze
HPA: Hypothalamus—pituitary—adrenal
i.p.: intraperitoneal
kHz: kilohertz
OFT: Open field test
RM: repeated measures
s: second
SAP: Spontaneous alternation percentage
SEM: standard error mean
US: unconditioned stimulus.
