# Supplementary material for: Exploring Refinement Strategies for Single Housing of Male C57BL/6JRj Mice: Effect of Cage Divider on Stress-Related Behavior and Hypothalamic-Pituitary-Adrenal-Axis Activity
Source: Front Behav Neurosci. 2021 Oct 28;15:743959. doi: 10.3389/fnbeh.2021.743959 (PMC8581484; doi:10.3389/fnbeh.2021.743959)
Supplement: Supplementary file 1 [file Data_Sheet_1.docx]

Supplementary Material

1. **Normalized body weight**


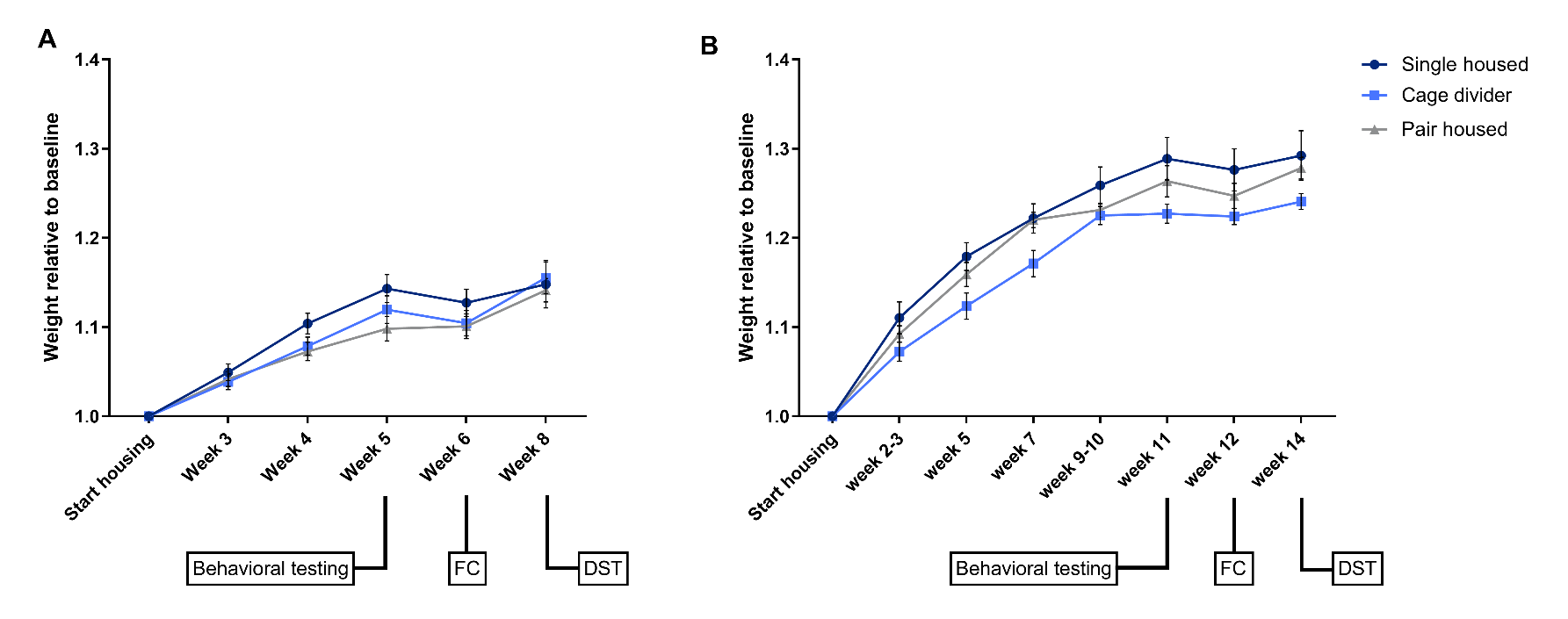
In both experiments, normalized body weights were not significantly different between the housing groups but increased over time (Exp. 1: Interaction: F_10,135_ = 1.173, *P* = 0.3142; Time effect: F_5,135_ = 109.7, *P* < 0.0001; Housing effect: F_2,27_ = 0.9545, *P* = 0.3976; **Supplementary Figure 1A** – Exp. 2: Interaction: F_14,189_ = 1.684, *P* = 0.0617; Time effect: F_7,189_ = 352.0, *P* < 0.0001; Housing effect: F_2,27_ = 3.222, *P* = 0.0556; **Supplementary Figure 1B**).

**Supplementary Figure 1: Normalized body weights of mice that are single housed, pair housed and pair housed with a cage divider.** **A)** Normalized body weights in experiment 1. **B)** Normalized body weights in experiment 2. Data are presented as mean ± s.e.m. *n* = 10 mice/housing condition. Statistical analysis: Repeated measures Two-way ANOVA. FC = fear conditioning; DST = dexamethasone suppression test.

1. **Open field test**

In experiment 1, the total distance traveled was not statistically different between the housing conditions in the time interval 0 – 5 (F = 3.073, *P* = 0.0627; **Supplementary** **Figure 2A**), while a significant housing effect was observed in the 5 – 10 time interval (F = 3.929, *P* = 0.0318; **Supplementary Figure 2B**). In the latter time interval, the total distance traveled was significantly lower in pair housed mice (p < 0.05) compared to mice housed with a cage divider. The time spent in the center zone during the first five minutes was not affected by housing (F = 0.05479, *P* = 0.9468; **Supplementary Figure 2D**), but was significantly different in the 5 – 10 min time interval (F = 4.301, *P* = 0.0239; **Supplementary Figure 2E**) between single housed and pair housed mice (*P* = 0.0215).

The total distance traveled was unaffected by housing in experiment 2 in either the 0 – 5 (F = 2.400, *P* = 0.1098; **Supplementary Figure 3A**) and 5 – 10 (F = 3.337, *P* = 0.0507; **Supplementary Figure 3B**) time interval, although a trend was present. Similarly, the time spent in the center zone was not statistically different between the housing conditions in the 0 – 5 (F = 2.313, *P* = 0.1183; **Supplementary Figure 3D**) and 5 – 10 (F = 0.7105, *P* = 0.5004; **Supplementary Figure 3E**) time interval.


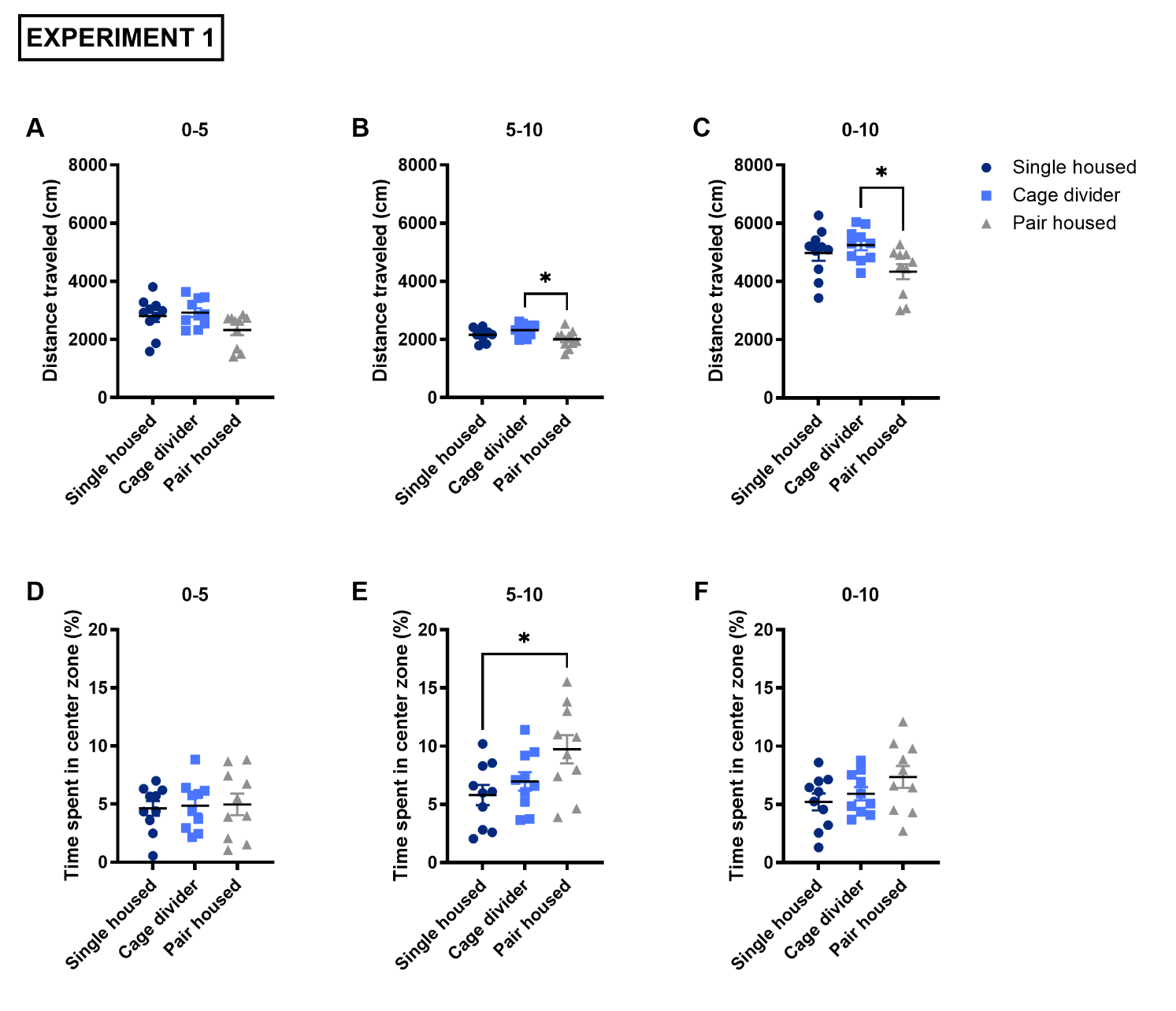
**Supplementary Figure 2: Extended analysis of the open field test in experiment 1. A)** Total distance travelled in time interval 0–5 min. **B)** Total distance travelled in time interval 5–10 min. **C)** Total distance travelled in time interval 0–10 min. **D)** Time spent in the center zone in time interval 0–5 min. **E)** Time spent in the center zone in time interval 5–10 min. **F)** Time spent in the center zone in time interval 0–10 min. Data are presented as mean ± s.e.m. *n* = 10 mice/housing condition. Statistical analysis: Ordinary One-way ANOVA with Tukey’s multiple comparisons test for comparisons between single housed, pair housed and mice housed with a cage divider. * *P* < 0.05.

**
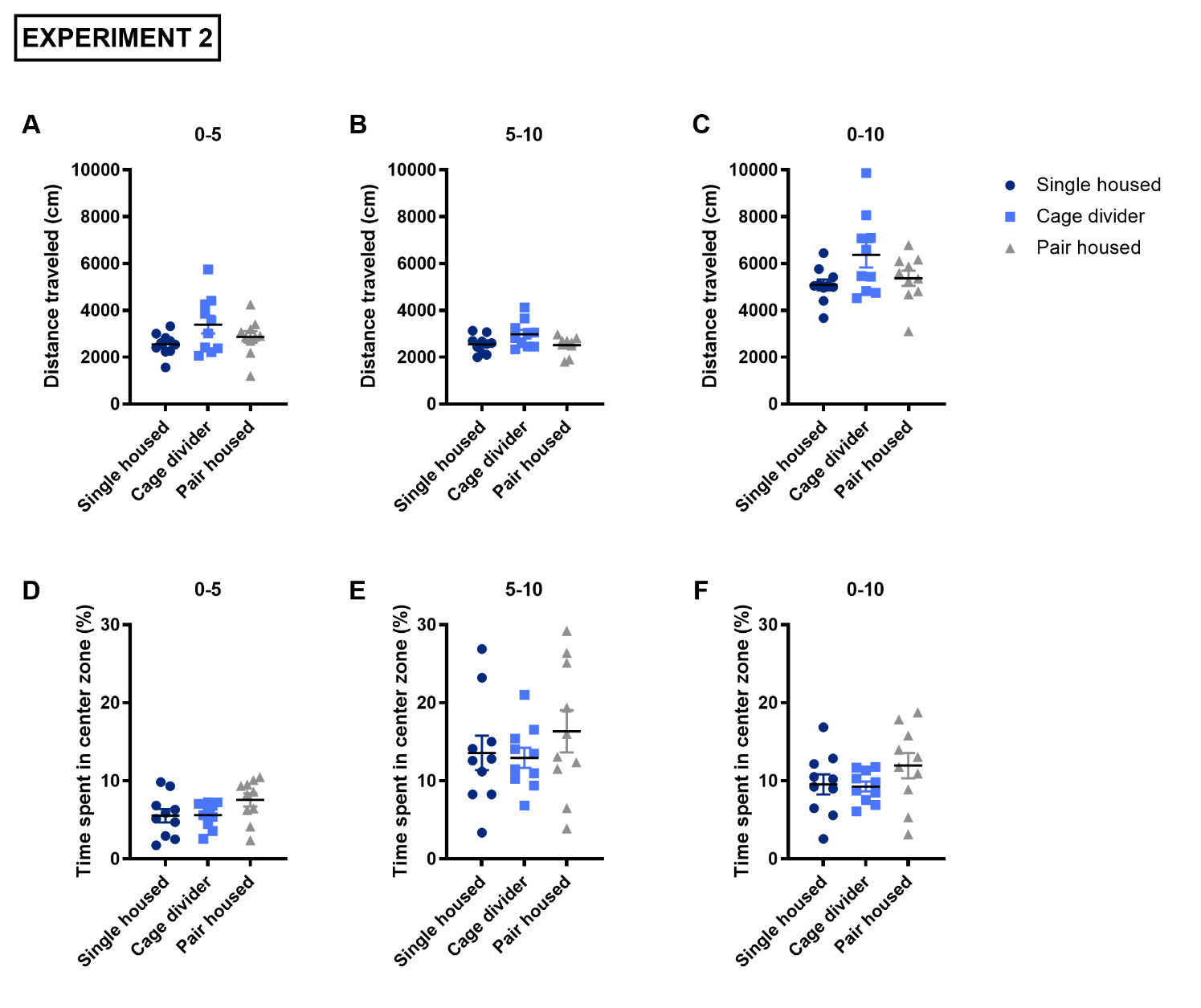
Supplementary Figure 3: Extended analysis of the open field test in experiment 2. A)** Total distance travelled in time interval 0–5 min. **B)** Total distance travelled in time interval 5–10 min. **C)** Total distance travelled in time interval 0–10 min. **D)** Time spent in the center zone in time interval 0–5 min. **E)** Time spent in the center zone in time interval 5–10 min. **F)** Time spent in the center zone in time interval 0–10 min. Data are presented as mean ± s.e.m. *n* = 10 mice/housing condition. Statistical analysis: Ordinary One-way ANOVA.

1. **
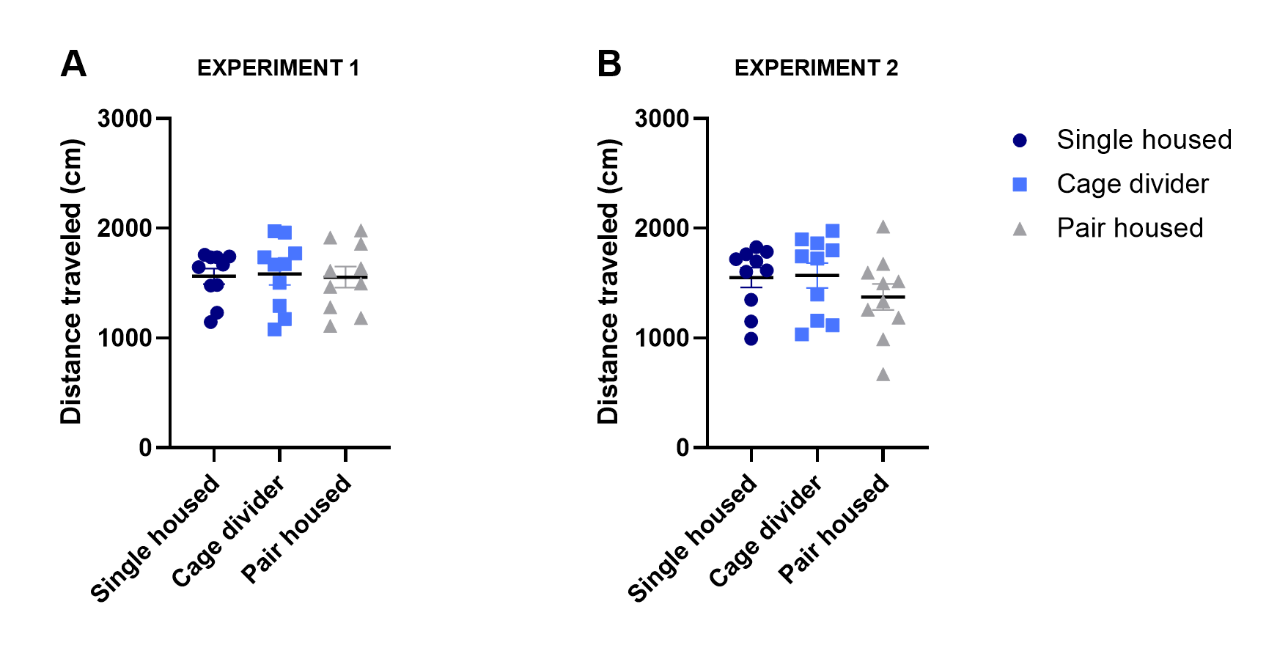
Elevated plus maze test**

**Supplementary Figure 4. Total distance traveled in the elevated plus maze test. A)** Total distance traveled in experiment 1. **B)** Total distance traveled in experiment 2. Data are presented as mean ± s.e.m. *n* = 10 mice/housing condition. Statistical analysis: Ordinary One-way ANOVA.

1. **Distance moved during HAB phase in the fear conditioning box**

**
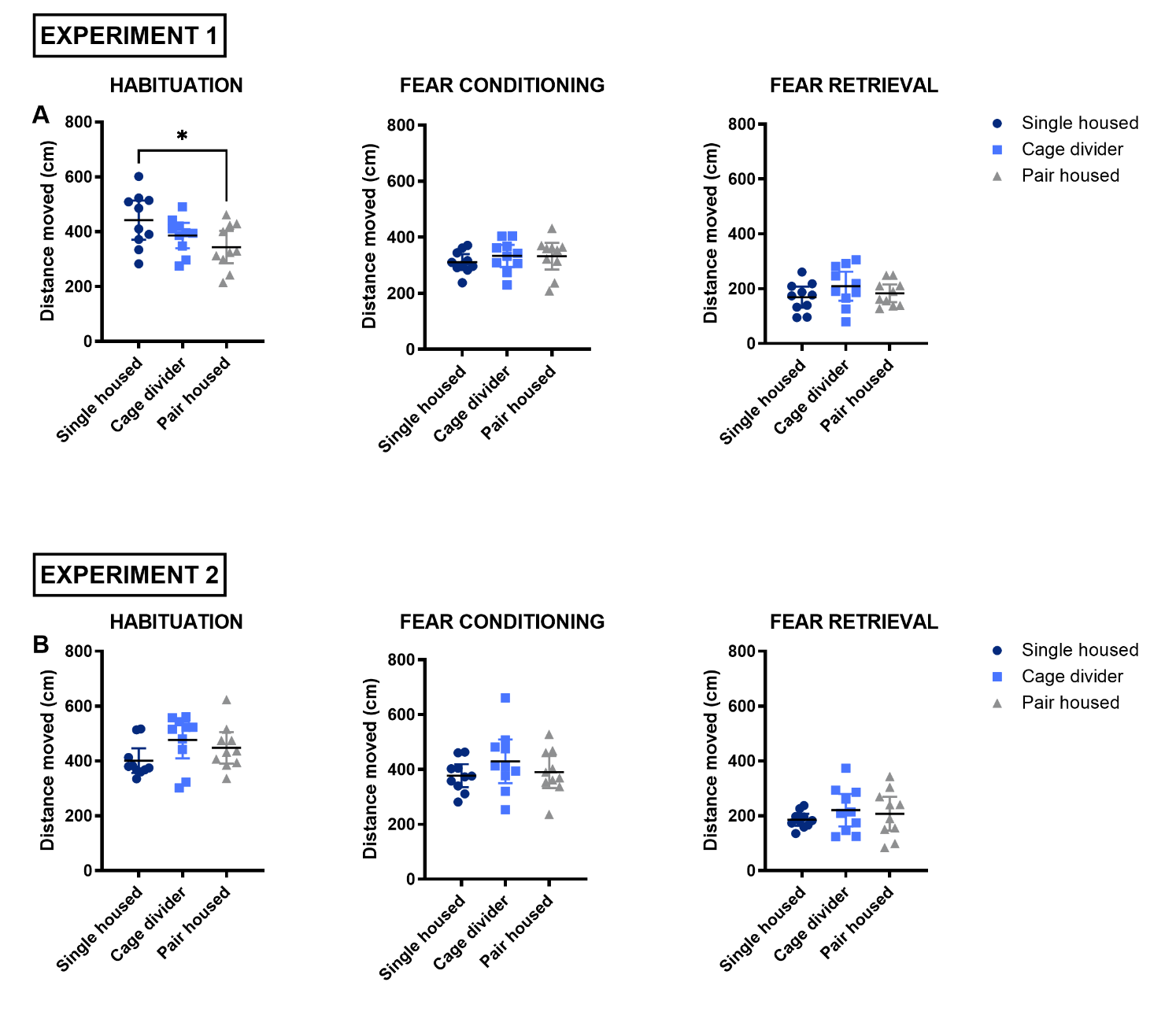
Supplementary Figure 5. Analysis of distance moved during the HAB phase in the fear conditioning box.** The graphs illustrate the total distance moved (cm) during the acclimation period (HAB) to the test box in the habituation, the fear conditioning and the fear retrieval test. **A)** Overview of distance moved during the HAB phase in experiment 1. **B)** Overview of distance moved during the HAB phase in experiment 2. Data are presented as mean ± s.e.m. *n* = 10 mice/housing condition. Statistical analysis: Ordinary One-way ANOVA with Tukey’s multiple comparisons test for comparisons between single housed, pair housed and mice housed with a cage divider. * *P* < 0.05.

|  | **Experiment 1** | | | **Experiment 2** | | |
| --- | --- | --- | --- | --- | --- | --- |
|  | **Single housed** | **Cage divider** | **Pair**  **housed** | **Single housed** | **Cage divider** | **Pair**  **housed** |
| Open field | 4973 ± 263.4 | 5248 ± 179.6 | 4335 ± 259.5 | 5088 ± 233.7 | 6368 ± 540.8 | 5372 ± 324.0 |
| *Distance traveled (cm)* |  |  |  |  |  |  |
| *Time spent in center zone (%)* | 5.222 ± 0.7261 | 5.926 ± 0.5640 | 7.364 ±0.9414 | 9.549 ± 1.278 | 9.274 ± 0.6429 | 11.95 ± 1.611 |
| Y-maze SAT | 63.41 ± 3.258 | 67.13 ± 1.859 | 64.45 ± 2.293 | 65.39 ± 3.690 | 64.76 ± 2.383 | 59.76 ± 1.102 |
| *SAP (%)* |  |  |  |  |  |  |
| *Arm entries (amount)* | 41.10 ± 1.588 | 41.10 ± 1.935 | 41.10 ± 2.350 | 37.6 ± 1.536 | 46.50 ± 2.262 | 37.80 ± 2.215 |
| EPM  *Time spent in open arms (%)* | 8.206 ± 1.571 | 8.502 ± 1.334 | 6.028 ± 1.472 | 5.634 ± 1.165 | 7.068 ± 1.451 | 8.761 ± 2.064 |
| Adrenal gland weight *(mg/g Body weight)* | 0.09744 ± 0.01236 | 0.08626 ± 0.01008 | 0.07553 ± 0.01064 | 0.05861 ± 0.01040 | 0.07733 ± 0.01531 | 0.09509 ± 0.01506 |
| Pituitary gland weight *(mg/g Body weight)* | 0.02492 ± 0.003113 | 0.02271 ± 0.003011 | 0.02555 ± 0.002602 | 0.01780 ± 0.001197 | 0.01960 ± 0.002599 | 0.02034 ± 0.003387 |
| Corticosterone  *Vehicle (ng/mL)*  *Dexamethasone (ng/mL)* | 77.34 ± 19.40 | 72.82 ± 16.08 | 100.2 ± 21.68 | 106.4 ± 18.44 | 76.49 ± 4.462 | 84.98 ± 4.237 |
|  | 13.85 ± 4.696 | 14.81 ± 5.327 | 24.24 ± 8.823 | 15.11 ± 13.36 | 39.15 ± 16.48 | 45.54 ± 13.55 |

**Supplementary Table 1: Descriptive statistics.** Data are presented as mean ± s.e.m. *n* = 10 mice/housing condition. EPM = Elevated Plus Maze; SAP = Spontaneous Alternation Percentage; SAT = Spontaneous Alternation Test.
